# Understanding the Percolation Effect in Triboelectric Nanogenerator with Conductive Intermediate Layer

**DOI:** 10.34133/2021/7189376

**Published:** 2021-02-04

**Authors:** Binbin Zhang, Guo Tian, Da Xiong, Tao Yang, Fengjun Chun, Shen Zhong, Zhiming Lin, Wen Li, Weiqing Yang

**Affiliations:** ^1^Key Laboratory of Advanced Technologies of Materials, Ministry of Education, School of Materials Science and Engineering, Southwest Jiaotong University, Chengdu 610031, China; ^2^College of Electronic and Information Engineering, Southwest University, Chongqing 400715, China

## Abstract

Introducing the conductive intermediate layer into a triboelectric nanogenerator (TENG) has been proved as an efficient way to enhance the surface charge density that is attributed to the enhancement of the dielectric permittivity. However, far too little attention has been paid to the companion percolation, another key element to affect the output. Here, the TENG with MXene-embedded polyvinylidene fluoride (PVDF) composite film is fabricated, and the dependence of the output capability on the MXene loading is investigated experimentally and theoretically. Specifically, the surface charge density mainly depends on the dielectric permittivity at lower MXene loadings, and in contrast, the percolation becomes the degrading factor with the further increase of the conductive loadings. At the balance between the dielectric and percolation properties, the surface charge density of the MXene-modified TENG obtained 350% enhancement compared to that with the pure PVDF. This work shed new light on understanding the dielectric and percolation effect in TENG, which renders a universal strategy for the high-performance triboelectronics.

## 1. Introduction

With the rapid evolution of artificial intelligence (AI), Internet of things (IoT), and portable and wearable electronics, there is a great challenge to develop a distributed, sustainable, and mobile power source for driving such electronic devices in these areas [1–4]. Recently, harvesting ambient mechanical energy such as wind, water, and human motion has been proved as an excellent way to provide energy for such sensing nodes [5–7]. Compared with an electromagnetic generator (EMG), the triboelectric nanogenerator (TENG) has emerged as a powerful platform for the transformation of ambient mechanical energy into electricity and possesses merits of high-efficiency, low-cost, environmental friendliness, reliable, and diverse choice of materials, which has attracted much attention in recent years [8–10]. However, a major challenge of the TENG is the relatively low surface charge density that limits the output performance and potential applications [11–15]. Many efforts have been made to improve the surface charge density, such as material selection [12], surface charge injection [13], tribomaterial surface modification [14–17], intermediate layer integration [18], external-charge pumping [19], and self-charge excitation [20, 21]. Among these methods, embedding conductive intermediate layer into TENG provides an efficient, cost-friendly, and scalable pathway to improve the surface charge density and output performance [18]. In previous works, metal (gold, silver, copper, and aluminum) and carbon materials (active carbon, graphene, and carbon black) were embedded in the polymer acted as the intermediate layer to enhance the dielectric permittivity of the polymer film, as a result, the output of TENG was significantly improved [22–25]. However, accompanied by an increase of the dielectric permittivity, percolation causes a leakage current between the dielectric membrane and the back electrodes, which is unfavorable for the output of the device. Such an effect plays a crucial role in the performance of the TENG while be ignored. Thus, a universal and fundamental theory including the dielectric effect and percolation remains highly desired.

Herein, we prepared a Ti_3_C_2_T*_x_* MXene-doped polyvinylidene fluoride (MXene/PVDF) composite film with highly enhanced triboelectric performance via tuning the content of MXene flakes as well as the balance between the dielectric property and the percolation effect. It is found that the dielectric permittivity of the composite film increases with the enhancement of the MXene doping content from 0% to 25% while the output of the TENG reaches a maximum at the doping content of 10%. This result can be explained by the fact of the existence of percolation. Employing experiment testing and theoretic simulation, it is indicated that the conductivity of the film follows the exponential relationship with the doping content of the MXene flakes. Percolation occurs at the doping content higher than 10% resulting in the tremendous leakage current between the dielectric film and the back electrodes. As a result, the surface charge density of the TENG drops especially at the high doping content. At the balance point with the doping content of 10%, the open-circuit voltage, surface charge density, and short-circuit current of the developed TENG employing the MXene/PVDF film are increased by 320%, 350%, and 610% compared to the original pure PVDF thin film. In summary, the current dielectric theory of TENG is enriched via introducing the percolation effect, this work not only paves a new way to understand the fundamental mechanism of TENG but also provides an efficient strategy for the output amplification of the triboelectronics.

## 2. Results and Discussion

### 2.1. Design and Synthesis of the MXene/PVDF Membrane

**Figure 1:**
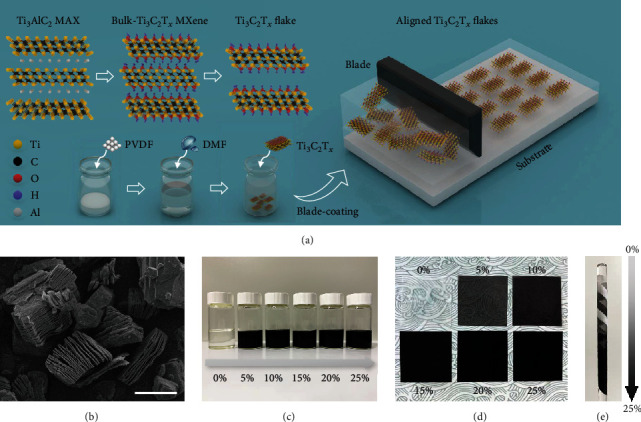
Design and synthesis of the MXene-embedded PVDF membrane. (a) Schematic illustration of the formation procedure of the Ti_3_C_2_T*_x_* MXene flakes (etching and ultrasonication) and the preparation of the MXene-embedded PVDF membrane by blade coating. (b) Scanning electron microscopy (SEM) image of the fabricated MXene flakes. Scale bar, 5 *μ*m. (c) Digital photographs of the MXene/PVDF composite solution with different doping content of MXene ranging from 0% to 25%. (d, e) Images of the prepared MXene-embedded PVDF membrane with different doping content.

As a new family of 2D materials, MXenes have recently attracted tremendous interest in research owing to their outstanding characteristics in numerous applications, including flexible electronics, energy storage, energy harvesting, and electromagnetic shielding, to name a few. Generally, MXene is fabricated by etching the “A” element from the relevant MAX phases and then possesses the structure of M_*n*+1_X*_n_*T*_x_*, where M denotes the transition metal, X represents carbon or nitrogen, and T*_x_* is the surface terminations including oxygen, hydroxyl, and fluorine. As schematically illustrated in Figure 1(a), in this work, Ti_3_C_2_T*_x_* MXene is fabricated by selectively etching the aluminum element from the MAX-phase Ti_3_AlC_2_. Hydrofluoric acid (HF) is chosen to be the etchant and ultrasonication is introduced to magnify the layer spacing and produce the MXene flakes. PVDF composite is prepared by adding the PVDF powder into N, N-dimethylformamide (DMF), and the homogenous solution is formed by continuous stirring at 70°C for 3 hours. Ti_3_C_2_T*_x_* MXene and PVDF mixture is prepared by adding the MXene flakes into the prepared solution, and the mass ratio between the MXene flakes and the PVDF powder is ranging from 5% to 25%. The terminal functional groups of -F, -OH on the surface are favorable of the MXene flakes' dispersion in the PVDF matrix. The MXene/PVDF composite film is fabricated via blade coating. The SEM image of the accordion-like MXene flakes is shown in Figure 1(b). As shown in Figure 1(c), as MXene mixing into the PVDF solution, the color of the mixture turns from transparency to black. A similar trend is also reflected in the fabricated MXene/PVDF composite film (as shown in Figures 1(d) and 1(e)). The transparency of the film drops with an increase of the MXene loading, from semitransparency to opaque.

### 2.2. Characterization of the MXene/PVDF Membrane

**Figure 2:**
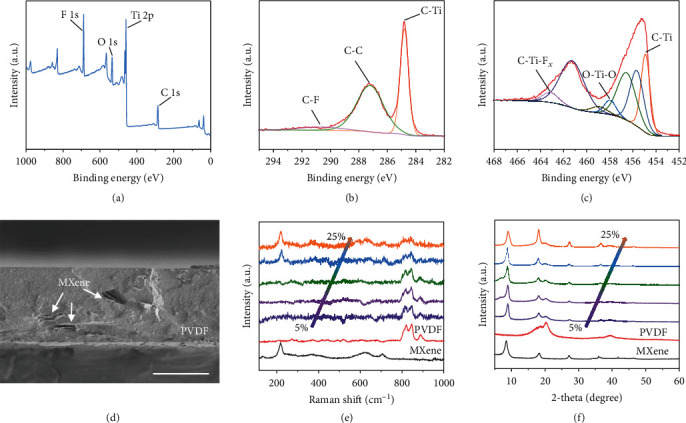
Characterization of the MXene/PVDF membrane. (a) X-ray photoelectron spectroscopy (XPS) survey spectra of the Ti_3_C_2_T*_x_* MXene. (b, c) C 1s (b) and Ti 2p (c) spectra of the MXene. (d) Cross-section view of the MXene/PVDF membrane with doping content of 25%. Scale bar, 30 *μ*m. (e) Raman spectra of the MXene, PVDF, and MXene/PVDF composite film with different doping content ranging from 5% to 25%. (f) X-ray diffraction (XRD) patterns of the MXene, PVDF, and MXene/PVDF composite film at different loadings.

X-ray photoelectron spectroscopy is employed to investigate the surface chemistry of Ti_3_C_2_T*_x_* MXene flake (as shown in Figures 2(a)–2(c)). The full survey reveals that F, O, Ti, and C are the main elements in the fabricated MXene flakes (as demonstrated in Figure 2(a)). The high-resolution C 1s spectra are illustrated in Figure 2(b), the presence of C-F, C-C, and C-Ti bands reveals that the Ti_3_C_2_ structures are well maintained after HF etching and fluorine group is presented on the MXene surface. As shown in Figure 2(c), the high-resolution Ti 2p spectra reveal that the presence of C-Ti, Ti-F, and Ti-O bands, moreover, the absence of Ti-Al band concludes that the aluminum atom layers are etched and replaced by the terminal functional groups such as fluorine, oxygen, and hydroxyl. To obtain the distribution status of the MXene flakes, a cross-section view of the composite film is captured by SEM (as shown in Figure 2(d)). It is indicated that the MXene flakes are arranged in parallel in the PVDF matrix that is beneficial for the improvement of the dielectric property, furthermore, the thickness of the film is 40 *μ*m. In addition, Raman spectroscopy and X-ray diffraction are utilized to analyze the composition and structure of the MXene/PVDF film. As shown in Figure 2(e), the A_1g_ (Ti, O, C) around 200 cm^−1^ and A_1g_ (C) around 720 cm^−1^ are detected in the pure Ti_3_C_2_T*_x_* MXene flakes, and the peaks located at around 820 cm^−1^ and 845 cm^−1^ are detected in pure PVDF. Besides, with the enhancement of the MXene loadings from 5% to 25%, the intensity of the A_1g_ (Ti, O, C) and A_1g_ (C) increase while the intensity of the peaks at 820 cm^−1^ and 845 cm^−1^ decreases, indicating that MXene flakes are successfully embedded in the PVDF matrix. The same trend is also reflected in the XRD patterns (as shown in Figure 2(f)), the intensity of the peak located in 8.5, 18, and 27.2 degrees increases with the enhancement of MXene loadings.

### 2.3. Dielectric and Percolation Properties of the MXene/PVDF Membrane

**Figure 3:**
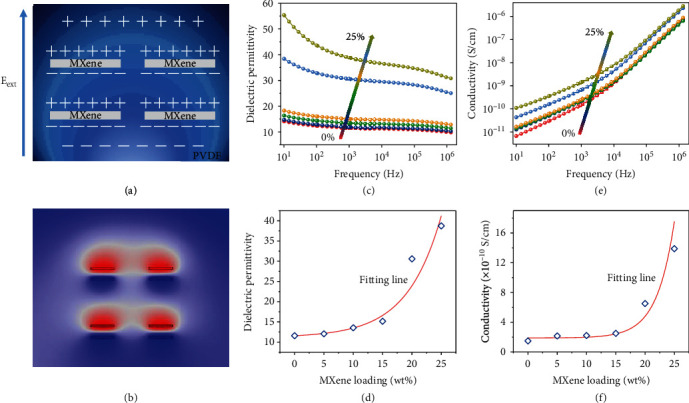
Dielectric and percolation properties of the MXene/PVDF membrane. (a) Schematic illustration of the dielectric effect in MXene/PVDF composite and the polarization charges distribution in the MXene/PVDF metal-insulator-metal (MIM) capacitors under the external electric field (E_ext_). (b) Corresponding simulation of the potential distribution on MXene flakes by COMSOL Multiphysics. (c) Dielectric permittivity of the MXene/PVDF composite versus MXene loading at various frequencies (10 Hz-10 MHz). (d) Dielectric permittivity of the MXene/PVDF composite at different loadings measured at 1 kHz. (e) Conductivity of the MXene/PVDF composite versus MXene loading at various frequencies (10 Hz-10 MHz). (f) Conductivity of the MXene/PVDF composite at different loadings measured at 1 kHz.

The dielectric model of the MXene/PVDF composite can be elaborated by the microcapacitor model [26, 27] (as schematically demonstrated in Figure 3(a)). Typically, metal-insulator-metal (MIM) capacitors form with the MXene embedding in the host PVDF, and interface polarization at the interfaces of the conductive filler and polymer foundation is established when an external electric field is applied on the composite film. The internal MIM capacitors further improve the dielectric property of the MXene/PVDF composite compared to that of the MXene-free PVDF film where the polarization charge is dispersed on the surface. Finite element analysis (FEA) is employed to simulate the potential distribution on the MIM microcapacitors, and the result is constant with the theory (as shown in Figure 3(b)). The dielectric properties of the MXene-free and MXene-embedded PVDF films are demonstrated in Figures 3(c)–3(f). As shown in Figure 3(c), the dielectric permittivity of the fabricated film increases with the enhancement of the MXene loadings from 0% to 25% while decreases with the increase of the electric field frequency. At the frequency of 1 kHz, the dielectric permittivity of the film improves from 11.58 to 38.75 when the MXene loading increases from 0% to 25% (as shown in Figure 3(d)). Besides, the conductivity of the film increases with the improvement of the MXene loadings and testing frequencies (as shown in Figure 3(e)). At the frequency of 1 kHz, the conductivity of the film increases from 1.49 to 13.89 × 10^−10^ S/cm when the MXene loading transfers from 0% to 25%. It is worth noting that there is a sharp turning point in the dielectric permittivity and conductivity at the loading of 15%, revealing the existence of percolation [28, 29]. At this turning point (percolation limit), the distance of the neighboring conductive fillers drops significantly resulting in a very thin insulator layer between the MXene flakes. In this case, the dielectric permittivity and conductivity of the MXene/PVDF composite film rise sharply.

### 2.4. Triboelectrification of the MXene/PVDF Membrane

**Figure 4:**
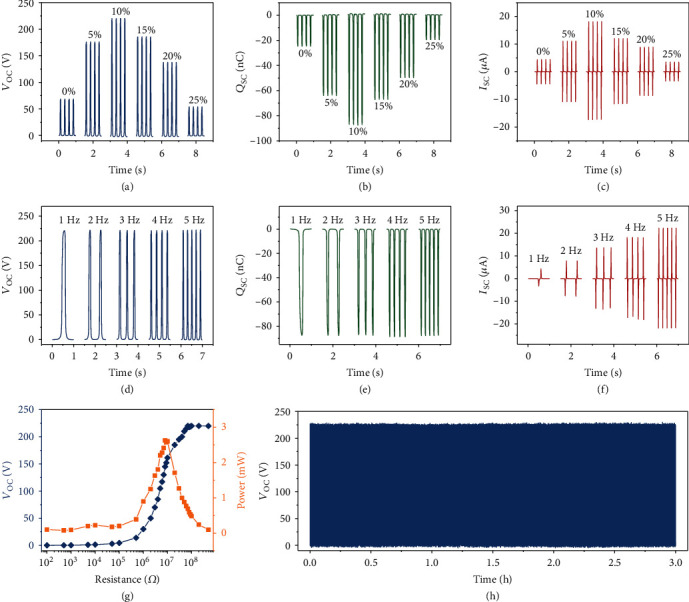
Triboelectrification of the MXene/PVDF membrane. (a–c) Open-circuit voltage (a), short-circuit transferred charges (b), and short-circuit current (c) of the TENG based on MXene/PVDF membrane at different loadings ranging from 0% to 25%. (d–f) Open-circuit voltage (d), short-circuit transferred charges (e), and short-circuit current (f) of the TENG at MXene loading of 10% operated at different frequencies (1-5 Hz). (g) Dependence of the output performance of TENG on the external loadings. (h) Stability test of the fabricated TENG based on MXene/PVDF membrane.

To study the dependence of dielectric and percolation properties on the output of the triboelectric nanogenerator, a contact-separation mode TENG is fabricated. As shown in Figures 4(a)–4(c), there are similar trends in the open-circuit voltage, short-circuit transferred charge, and short-circuit current of the TENG, that is, increase with the MXene loadings rising from 0% to 10% and then decrease with the loadings rising from 10% to 25%. At optimal MXene loading of 10%, the open-circuit voltage, short-circuit transferred charge, and short-circuit current are 220 V, 87.5 nC, and 18 *μ*A, respectively. Compared to that of the TENG fabricated by the pure PVDF membrane, there are 323%, 354%, and 410% enhancements in the open-circuit voltage, short-circuit transferred charge, and short-circuit current. This phenomenon is associated with the balance of the dielectric and percolation properties. In detail, the enhancement of the dielectric permittivity creates more electron traps in the composite film which is beneficial for the output of the TENG, while the increase of the percolation induces more leakage current between the dielectric film and the back electrodes which harms the output. Moreover, the output of the TENG at MXene loading of 10% at different operation frequencies is illustrated in Figures 4(d)–4(f). It is indicated that the open-circuit voltage and short-circuit transferred charge of the TENG is constant with the operation frequency, while the short-circuit current increases with the rising of the frequency from 1 Hz to 5 Hz. Dependence of the output of the TENG on the external resistances is also investigated, as shown in Figure 4(g), the open-circuit voltage increases with the increase of the external loadings, and the output power reaches a maximum of 2.6 mW at the loading of 8 M*Ω*. Also, the stability of the fabricated TENG is tested, as shown in Figure 4(h), the open-circuit voltage shows imperceptible drops after continuous operating for 20,000 times at an operating frequency of 2 Hz.

### 2.5. Dielectric and Percolation Effect in the TENG

**Figure 5:**
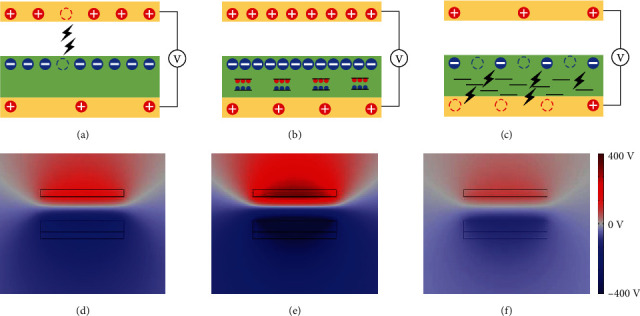
Dielectric and percolation effect in the TENG. (a–c) Current carrier distribution and recombination in TENG based on pure PVDF film (a), low-concentration MXene-modified PVDF film (b), and high-concentration MXene-modified PVDF film (c). Yellow layer: conductive layer. Green layer: dielectric layer. Black line: conductive filler. (d–e) Numerical calculations on the potential distribution in TENG based on pure PVDF film (d), low-concentration MXene-modified PVDF film (e), and high-concentration MXene-modified PVDF film (f).

To further understand the dielectric and percolation effects in the TENG, based on the above results, the potential mechanism is proposed in Figure 5. First, for the working principle of TENG, the electrons transfer from the conductive layer to the dielectric layer when they contact with each other owing to the different electron affinity of the two materials. As a result, the dielectric layer is negatively charged and the conductive layer is positively charged. Besides, these charges keep sustained on the surface and unable to be neutralized for a while. The output of the TENG mainly depends on the surface charge density of the dielectric film, and based on Paschen's law, the maximum surface charge density is decided by the air breakdown limit (as shown in Figure 5(a)). To enhance the air breakdown limit, improving the dielectric permittivity is a basic and efficient way, for instance, forming the conductive intermediate layer in the dielectric foundation. As shown in Figure 5(b), microcapacitors form when the conductive filler adding in the insulator layer and more electrons can be trapped, as a result, the air breakdown limit and surface charge density are significantly enhanced. With continuous adding the conductive filler in the dielectric layer, the distance of the conductive filler drops. In addition, electrical breakdown occurs between the dielectric layer and the back electrodes, resulting in the reduction of the total surface charge (as shown in Figure 5(c)). Especially, the surface charge density and output of the TENG drop sharply when the loading of the conductive filler exceeds the point of percolation limit. The corresponding potential distribution on the dielectric layer and the electrodes are simulated by the finite element analysis, which is consistent with the mechanism (as shown in Figures 5(d)–5(f)).

## 3. Conclusion

In summary, the MXene-embedded PVDF film with high dielectric permittivity was fabricated via blade-coating and acted as the functional layer in the TENG. We systematically investigated the contribution of the MXene filler in the dielectric properties of the film and the output performance of the fabricated TENG. Based on the experimental and theoretical results, we concluded that the dielectric permittivity of the film played a foremost role in the surface charge density of TENG when the conductive filler loading is below the percolation limit, while the percolation effect became more and more important in the surface charge density with the enhancement of the filler loading, especially above the percolation limit. In this work, at the optimal MXene loading of 10%, the surface charge density of the composite film increased by 350% compared to that of the pure insulator film. In future works, for acquiring an optimal output of the TENG, enhancement of the dielectric permittivity and meanwhile inhibition of the percolation of the charges should be considered.

## 4. Materials and Methods

### 4.1. Synthesis of the Ti_3_C_2_T*_x_* MXene Flakes

The multilayer Ti_3_C_2_T*_x_* was synthesized by etching Ti_3_AlC_2_ MAX phase via the etchant of HF. In detail, the 2.5 g Ti_3_AlC_2_ power was stirred magnetically with 25 ml 40 wt% HF to break the Ti-Al metal bonds at 40°C for 24 h. After that, the fabricated product was repeatedly washed with deionized water to remove the residual HF until the pH value was over 6. Then, the gotten 90 mg Ti_3_C_2_T*_x_* MXene power was dispersed into 30 ml deionized water with ultrasonication treatment for 30 min and then centrifuged at 3500 rpm for 1 hour. The upper suspension was collected and filtered through a polypropylene filter, followed by vacuum drying at 40°C for 24 h.

### 4.2. Fabrication of the Ti_3_C_2_T*_x_* MXene-Embedded PVDF Composite Film

The MXene/PVDF composite film was fabricated by the blade-coating method. For the preparation of the solution, in detail, PVDF solution with 15 wt% in DMF was prepared by stirring at 70°C for 3 h. Then, the prepared MXene powder was added to the PVDF solution and stirred magnetically at room temperature for 24 h. The mass ratio between the MXene and PVDF was ranging from 5% to 25%.

### 4.3. Fabrication of the TENG Device

Two pieces of 30 by 30 mm acrylic plates were cut from a 2.0 mm thick acrylic sheet and acted as the substrate by laser cutting, upon which PU foam with a height of 2 mm was attached and acted as the buffer layer. Two pieces of copper tapes were attached to the acrylic substrate as the electrodes and the tribolayer. On one of the cooper tapes, the prepared dielectric film was attached. Finally, these two parts were connected by the Kapton bend.

### 4.4. Characterization

Scanning electron microscopy (JSM 7800F) was utilized to capture the morphology of the MXene flakes and MXene/PVDF composite film. X-ray photoelectron spectroscopy (ESCALAB XI +) was used to characterize the surface chemistry of the MXene. Raman spectra (HORIBA Jobin-Yvon XploRA ONE, 532 nm laser) and X-ray diffraction (Empyrean) were utilized to study the composition and structure of the MXene/PVDF film. The TENG was driven by the linear motor (LinMot H01 − 23 × 86/160). The open-circuit voltage, short-circuit current, and transferred charges were measured by using a Keithley 6514 electrometer and a data acquisition (National Instruments, BNC-2120).

## Data Availability

The data used to support the findings of this study are available from the corresponding author upon reasonable request.
